# Blueprinting of Assessment for the Lower Limb in Undergraduate Anatomy Curriculum

**DOI:** 10.7759/cureus.49357

**Published:** 2023-11-24

**Authors:** Nikha Bhardwaj, Hare Krishna, Surajit Ghatak, Kuldeep Singh

**Affiliations:** 1 Department of Anatomy/Department of Medical Education and Technology, All India Institute of Medical Sciences, Jodhpur, IND; 2 Department of Anatomy, All India Institute of Medical Sciences, Jodhpur, IND; 3 Department of Pediatrics/Department of Medical Education and Technology, All India Institute of Medical Sciences, Jodhpur, IND

**Keywords:** curriculum, anatomy, assessment, blueprint, medical education

## Abstract

Background: Competency-based medical education is followed by most of the medical schools in India. Health universities are changing their question paper pattern and practical assessment pattern to ensure valid and reliable evaluation of student learning but assessment of entire curriculum competencies is a difficult task to achieve. Assessment blueprinting can provide a guiding map for balanced and rational assessment. It ensures similar exam content from year to year (over- or under-representing a topic can be avoided). The assessment blueprint provides ideas of appropriate formats for the knowledge/skills being assessed. The study aims to observe facilitators' perceptions and develop an assessment blueprint for the anatomy curriculum.

Methods: This educational mixed-method study was conducted in the Department of Anatomy of All India Institute of Medical Sciences, Jodhpur, India. Perceptions of 13 facilitators of the Anatomy Department about assessment blueprinting were observed on a five-point Likert scale. Topics of the lower limb were identified based on the Undergraduate Curriculum Volume-1 document provided by the National Medical Council of the Government of India. Weightage to each topic was given on the basis of anatomical importance and clinical significance at the undergraduate level. Marks for the topic as per relative weightage were calculated. The blueprint was prepared and validated by the subject expert of the institute. An example question paper template was prepared as per the assessment blueprint.

Results: In the present study, all 13 (100%) participants agreed that there is a need for a valid assessment framework to maintain the reliability of assessment, 11 (85%) participants agreed that an assessment blueprint can provide a valid tool for assessment, and 10 (77%) participants agreed that assessments are not planned as per learning domain requirement, and planning of assessment as per blueprint requires more time and resource. Of the participants, eight (62%) agreed that all topics or competencies are not included while setting the question paper. The blueprint for assessment of the lower limb provided an idea of high-weightage and low-weightage topics and the suitable types of questions for these topics.

Conclusion: Assessment is the key element of competency-based medical education. For a complete, valid, and reliable assessment, a blueprint for assessment is the need of the hour. Assessment blueprinting in this study showed that the anterior thigh region, hip joint, knee joint, and vascular and nerve supply of the lower limb are topics with maximum weightage for the lower limb in the medical anatomy undergraduate curriculum. It can help in reducing under or over-representation of content. With the help of a blueprint, the examiners can assess the majority of content without any intentional or unintentional bias.

## Introduction

Competency-based medical education is followed by most of the medical schools in India. The entire medical curriculum is subdivided into topic-based competencies for each subject as per the National Medical Commission of India guidelines. The anatomy curriculum is subdivided into 82 topics and 409 competencies. Teaching-learning activities of medical undergraduates are based on these competencies. However, when it comes to the assessment of these competencies, there is a wide gap [1].

Currently, in our health professional education system, the question paper setter is one person, the examiner or assessor is another person, and the topic is taught by someone else. Although there are guidelines for the number and types of questions, there is no clear roadmap for the assessment of the entire curriculum. We usually observe an imbalance in assessment in the form of personal preferences, less or non-representation of some topics in assessment, oversimplification, and assessment with invalid tools [2,3].

In competency-based medical education, learning outcome is the key and the entire process is built around it. For the evaluation of outcomes, a robust, structured, and authentic assessment tool is required for the curriculum of each subject [1-3]. Blueprinting for assessment can help in communicating expectations to other stakeholders (e.g., examiners and academic administrators). One of the benefits of an assessment blueprint is that it can help students in their study planning [2]. In simple words, an assessment blueprint provides a guiding template based on the weightage of topics for overall and effective assessment. It can help in planning, teaching, and learning activities accordingly.

## Materials and methods

This educational mixed-method study was conducted in the Department of Anatomy, All India Institute of Medical Sciences (AIIMS), Jodhpur, India after obtaining ethical approval from the Institutional Ethics Committee, AIIMS, Jodhpur (AIIMS/IEC/2022/4100). For this study, 13 facilitators (eight males and five females) of the Department of Anatomy were asked about the need for a blueprint for the assessment of the lower limb curriculum. A five-point Likert scale was used for the facilitator's perception of the assessment. A perception questionnaire was prepared and validated by the members of the Department of Medical Education & Technology, AIIMS, Jodhpur. The perception questionnaire was introduced and perception data were recorded on a five-point Likert scale. Data were analyzed and measured in percentage.

Topics were selected as per the competency table of the undergraduate curriculum of the lower limb as per the guidelines of the competency-based undergraduate curriculum for the Medical Graduate 2018, Medical Council of India [1]. Weightage to each topic was given based on the anatomical importance and clinical significance (at the undergraduate level) of each topic. Anatomical importance and clinical significance were sub-categorized into three: (a) three points are given for the topic of maximum anatomical importance/clinical significance; (b) two points to relatively less important/significant; and (c) one point to comparatively least important/significant.

The weightage of each topic was calculated by multiplying points of anatomical importance with clinical significance. Based on the weightage of each topic, the possible marks distribution for the lower limb topics was decided. The blueprint was prepared and validated. Based on the blueprint of assessment, a sample question paper was prepared and validated by a subject expert.

For the validation of the assessment blueprint, five subject experts were asked to grade each item (topic and its weightage) on a scale of 1 to 4. Grade 4 means it is appropriate, 3 means appropriate with minor corrections, 2 means needs major correction, and 1 means not appropriate. The content validity index (CVI) was calculated for each item. Items with a CVI of 0.79 and above were included in the assessment blueprint [4].

## Results

**Figure 1:**
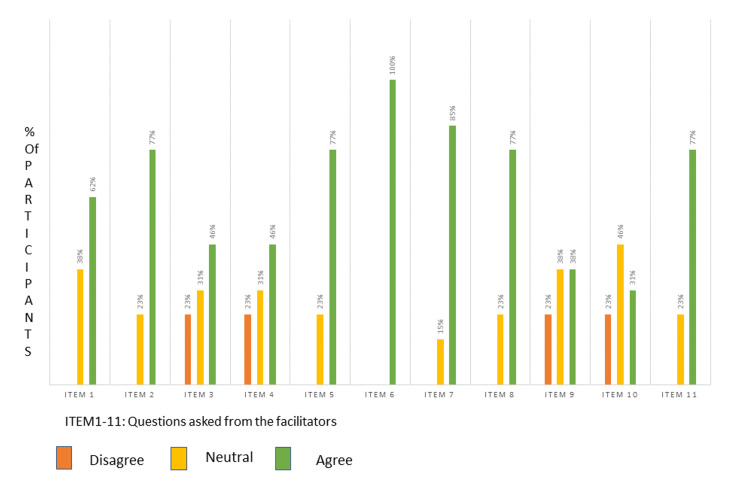
Graph showing facilitators' perception (%) of the need for an assessment blueprint

In this study, we observed the findings of 13 facilitators' perceptions of current assessment practices with the help of a five-point Likert scale (Table 1). We observed that 13 (100%) participants agreed that there is a need for a valid assessment framework to maintain the reliability of the assessment, and 11 (85%) participants agreed that the assessment blueprint can act as a valid tool for assessment (Figure 1). We also found that 10 (77%) participants agreed that assessments are not planned as per learning domain requirement, there are chances of unintentional bias during question paper setting, planning of assessment as per blueprint will require more time and resources, and implementation of blueprinting for assessment needs administrative support. Eight (62%) of the participants agreed that all topics or competencies are not included in the question paper setting (Figure 1). Six (46%) participants agreed that there are chances of intentional bias during the question paper setting (Figure 1).

**Table 1:** Facilitators' perception of the need for an assessment blueprint

S. No.	Item	Strongly disagree = 1	Disagree = 2	Neutral = 3	Agree = 4	Strongly agree = 5
1	Usually, all topics are not represented adequately in the question paper			5	5	3
2	Assessments are not planned as per the learning domain requirement			3	9	1
3	Teaching learning activities are aligned with the assessment		3	4	6	
4	There are chances of intentional bias during question paper setting	1	2	4	5	1
5	There are chances of unintentional bias during question paper setting			3	9	1
6	There is a need for a valid assessment framework to maintain the reliability of the assessment				8	5
7	An assessment blueprint can provide a valid tool for assessment			2	8	3
8	Planning of assessment as per the blueprint will require more time and resource			3	7	3
9	Blueprinting will help in the effective planning of the teaching-learning session		3	5	5	
10	Blueprinting will help students in their learning		3	6	4	
11	Implementation of blueprinting for assessment needs administrative support			3	6	4

There is a mixed response about the effect of blueprinting on student learning in which three (23%) disagreed, four (31%) agreed, and six (46%) showed a neutral/cannot say response (Figure 1).

This assessment blueprint was prepared for the lower limb anatomy (Table 2). A total of 18 topics were selected for the lower limb curriculum. Anatomical importance (I) and clinical significance (S) were subdivided into three and numbered from one to three on the basis of lower to higher importance/significance. Three points were given for the topic of maximum anatomical importance/clinical significance, two points for relatively less important/significant, and one point for comparatively least important/significant (this anatomical importance or clinical significance was relative and points were given on the basis of comparison with other topics).

**Table 2:** Assessment blueprint for the lower extremity/lower limb

Topic	Anatomical importance (I)	Significance (S)	W = (I × S)	Weightage (W/T)	Marks out of 100 (W/T × 100)	Marks out of 20
Front of thigh/anterior thigh region	3	3	9	9/96=0.09375	9.375	1.875~2
Gluteal region	2	3	6	6/96=0.0625	6.25	1.25~1
Back of thigh/posterior thigh region	1	2	2	2/96=0.0208	2.08	0.416~0.5
Hip joint	3	3	9	9/96=0.09375	9.375	1.875~2
Popliteal fossa	3	2	6	6/96=0.0625	6.25	1.25~1
Front of leg/anterior compartment of the leg	1	2	2	2/96=0.0208	2.08	0.416~0.5
Lateral compartment of the leg	1	1	1	1/96=0.0104	1.04	0.208~0.5
Back of leg/posterior compartment of the leg	2	1	2	2/96=0.0208	2.08	0.416~0.5
Knee joint	3	3	9	9/96=0.09375	9.375	1.875~2
Planter aspect of foot/sole	2	3	6	6/96=0.0625	6.25	1.25~1
Ankle joint	2	3	6	6/96=0.0625	6.25	1.25~1
Small joints of the foot	1	1	1	1/96=0.0104	1.04	0.208~0.5
Nerves	3	3	9	9/96=0.09375	9.375	1.875~2
Arteries of the lower limb	3	3	9	9/96=0.09375	9.375	1.875~2
Veins of lower limb	3	3	9	9/96=0.09375	9.375	1.875~2
Lymph nodes and lymphatic drainage of lower limb	1	2	2	2/96=0.0208	2.08	0.416~0.5
Arches of foot	3	2	6	6/96=0.0625	6.25	1.25~1
General feature, development, dermatome of lower limb	1	2	2	2/96=0.0208	2.08	0.416~0.5
			Total=96			20.5~20

Weightage of the each topic was calculated by multiplying anatomical importance with clinical significance.

On the basis of the anatomical importance and clinical significance of the above-said lower limb topics, a sample question paper was proposed in this study (Table 3).

**Table 3:** Sample question paper template for the lower limb MCQ: multiple choice question; VSAQ: very small answer question; SAQ: small answer question; PBQ: problem-based question. * 0.5 marks can be adjusted by merging two topics in the form of a clinical vignette-based MCQ.

Topic	Type of questions					Marks (total = 20)
	MCQ (0.5) marks each)	VSAQ (enumeration/anatomical basis) (01 marks each)	SAQ/clinical or embryological correlation (02 marks each)	Problem/case-based questions (02 marks each)	MLAQ (05 marks each)	
Front of thigh/anterior thigh region			1 question			2
Gluteal region		1 question				1
Back of thigh/posterior thigh region	1 question					0.5
Hip joint			1 question			2
Popliteal fossa		1 question				1
Front of leg/anterior compartment of the leg	1 question					0.5
The lateral compartment of the leg	1 question					0.5
Back of leg/posterior compartment of the leg	1 question					0.5
Knee joint				1 question		2
Planter aspect of foot/sole	2 questions					1
Ankle joint		1 question				1
Small joints of the foot	1 question					0.5
Nerves of the lower limb			1 question			2
Arteries of the lower limb	2 questions	1 question				2
Veins of the lower limb			1 question			2
Lymph nodes and lymphatic drainage of the lower limb	1 question					0.5
Arches of foot		1 question				1
General feature, development, dermatome of lower limb	1 question					0.5
Total items - 21 items (11 MCQ questions, 5 VSAQ, 4 SAQ, 1 PBQ)						Total=20.5*

## Discussion

Blueprinting is a very important tool that helps us in the alignment of curriculum with assessments. It also ensures the identification of high-weightage topics to prepare teaching learning sessions, formative assessments, and other educational activities of the department [2,5,6].

In the present study, there was complete agreement (100%) among participants about the need for valid assessment tools to ensure the reliability of the assessment. Approximately 77% of study participants believed that without an assessment blueprint, there are chances of unintentional bias like under-representation or over-representation of certain topics.

Raymond et al. developed a practical guide to test blueprinting. They subdivided the development of test blueprinting into four stages: stage 1 identifies major knowledge and skill domains; stage 2 delineates the assessment objective; stage 3 decides on the assessment format; and stage 4 specifies category weight [5].

Boulet et al. reported that the development of a method for assessment depends on two factors: the purpose of the assessment and the skills that are required to be tested [6]. Patil et al. reported that the blueprint aligns the objective with the assessment, and it guides examiners in a question paper setting. In their study, 89% of the faculty agreed that topic-wise question distribution was appropriate. They also recommended blueprinting as an integral part of assessment [7]. Ahmad and Hamed observed that the use of a blueprint for exams increased student achievement of learning objectives [8].

Goyal et al. reported that blueprinting guides paper construction and helps in the alignment of assessment with learning objectives. They also suggested that this assessment tool will help in the assessment of students' in-depth knowledge [9]. Ismail et al. used seven steps for the development of a blueprint. They also focused on possible challenges encountered during the assignment of weightage to the content [10].

Bridge et al. reviewed methods for the development of content-valid assessment tools in 144 medical schools about test blueprinting and reported that test blueprinting is important [11]. Tabish reported that the identification of the stakes involved is essential for planning and framing assessment tools [12].

Based on the weightage of the topic through the assessment blueprint, all learning domains like knowledge, skill, and attitude can be better assessed by specific methods like multiple choice questions, problem-based questions, and reasoning [13].

The current study developed an assessment blueprint for 18 topics of the lower limb anatomy curriculum. It was observed that six topics were high-weightage topics, seven were low-weightage topics, and the rest were average-weightage topics. The blueprint of the assessment can act as a guiding tool in constructing questions from the eligible topics of the lower limb. We know that the major threats of assessment are validity and construction under-representation [14,15]. It will help in reducing under or over-representation of content. With the help of a blueprint, the examiners can assess the entire curriculum without any intentional or unintentional bias. As shown in the example question paper, the incorporation of different types of questions will not only ensure assessment of the entire curriculum of that region but also help in testing higher domains.

Individuals learn in different ways and gain information accordingly. Learning styles affect study duration and also affect the scoring of a theoretical anatomy course [16]. If a blueprint for assessment is provided to the students at the beginning of the session, they can study at their own pace according to their learning styles.

Some people can say that blueprinting makes everything too predictive so it can hamper the development of analytical skills. This study suggests the assessment blueprinting only provides guidelines for question paper setting to ensure overall assessment. The type of question can be made different by merging two or more topics, splitting questions into subsections, and simultaneous planning for the practical examination. Success in any educational activity depends on effective and complete assessment, and blueprinting of the assessment can be an important pillar of it.

Limitations of the study

In this study, the need for the assessment blueprint was assessed only by intra-institute facilitators. Weightage of the topics, given based on anatomical importance and clinical significance, are comparative, so it may vary from observer to observer.

## Conclusions

Blueprinting of assessment should be an integral part of the medical examination system for valid and reliable assessment. The blueprint of the assessment provides an idea of the high-weightage and low-weightage topics, which can help in the selection of the type and number of questions required for a valid and reliable assessment of the given curriculum. A similar blueprint can be prepared for other areas of the anatomy curriculum.
